# Is Twelve Hours Really the Optimum Photoperiod for Promoting Flowering in Indoor-Grown Cultivars of *Cannabis sativa*?

**DOI:** 10.3390/plants12142605

**Published:** 2023-07-10

**Authors:** Ashleigh Ahrens, David Llewellyn, Youbin Zheng

**Affiliations:** School of Environmental Sciences, University of Guelph, Guelph, ON N1G 2W1, Canada; aahrens@uoguelph.ca (A.A.); dllewell@uoguelph.ca (D.L.)

**Keywords:** medicinal cannabis, daylength, flower initiation, controlled environment agriculture, lighting

## Abstract

*Cannabis sativa* (“cannabis” hereafter) is a valuable recent addition to Canada’s economy with the legalization for recreational use in 2018. The vast majority of indoor cannabis cultivators use a 12-h light/12-h dark photoperiod to promote flowering. To test the hypothesis that robust flowering initiation responses can be promoted in indoor-grown cannabis cultivars under longer photoperiods, clones of ten drug-type cannabis cultivars were grown under six photoperiod treatments. All treatments were based on a standard 24-h day and included 12 h, 12.5 h, 13 h, 13.5 h, 14 h, and 15 h of light. The plants were grown in a growth chamber for 3 to 4 weeks, receiving an approximate light intensity of 360 µmol·m^−2^·s^−1^ from white LEDs. Flowering initiation, defined as the appearance of ≥3 pairs of stigmas at the apex of the primary shoot, occurred in all cultivars under all photoperiod treatments up to 14 h. Delays in flowering initiation time under 14 h vs. 12 h ranged from no delay to approximately 4 days, depending on the cultivar. Some cultivars also initiated flowering under 15 h, but floral tissues did not further develop beyond the initiation phase. Harvest metrics of some cultivars responded quadratically with increasing photoperiod, with ideal levels of key flowering parameters varying between 12 h and 13 h. These results suggest there is potential to increase yield in some indoor-grown cannabis cultivars by using longer than 12-h photoperiods during the flowering stage of production. This is attributed to the inherently higher daily light integrals. Indoor cannabis growers should investigate the photoperiod responses of their individual cultivars to determine the optimal photoperiod for producing floral biomass.

## 1. Introduction

*Cannabis sativa* has been grown worldwide for food and fiber (i.e., hemp) and for the medicinal (e.g., cannabidiol, CBD) and psychoactive (e.g., Δ^9^-tetrahydrocannabinol, THC) effects of its secondary metabolites, which are particularly concentrated in the inflorescence tissues of female plants. Drug-type cannabis cultivars are defined as containing more than 0.3% THC and are commonly grown in indoor environments for security and crop uniformity purposes.

Plants in the cannabis genus are generally characterized as having short-day photoperiod responses (Zhang et al., 2021) [1], whereby reductions in daylength to a certain timeframe provoke flowering responses. However, cannabis’ widespread cultivation and breeding across many geographic regions have naturally led to substantial genotypic differences in photoperiodic responses (Clarke and Merlin, 2016; Zhang et al., 2018) [2,3]. These varying photoperiod responses have been exploited in different cultivation scenarios. For example, some hemp cultivars grown at higher latitudes have been bred to only initiate flowering at relatively short photoperiods, preventing flowering before harvest and maximizing vegetative growth (e.g., fiber yield) (Hall et al., 2014) [4]. In contrast, some drug-type cultivars have arisen from breeding with equatorial genotypes, where the minimal temporal changes in daylength are a relatively weak signal for seasonality. These cultivars tend to transition to flowering based on physiological age rather than photoperiod (i.e., day-neutral or “autoflowering”) (Small, 2022) [5].

Drug-type cannabis (“cannabis” hereafter) is commonly grown in indoor cultivation systems where electrical lighting sources provide 100% of the assimilation lighting. Indoor cultivation has higher infrastructure and energy costs than outdoor or greenhouse production, but growers have complete control of the cultivation environment, including the photoperiod. This fosters consistent production cycles in terms of length, yield, and quality. Since the main goal of cannabis production in indoor facilities is the production of floral tissues that are rich in secondary metabolites, a key production result is the consistent provocation of strong flowering responses. Indoor-grown cannabis cultivation normally begins with a period of vegetative growth (e.g., from germination or cloning stages to the end of the transplant stage) under long photoperiods (i.e., ≥16 h). Once plants have reached an appropriate size, they are transitioned to the flowering stage by invoking a short-day photoperiod, usually 12 h (Zheng and Llewellyn, 2022) [6]. The use of a 12-h photoperiod has become widespread in the indoor cannabis cultivation industry due to its ability to consistently induce rapid and robust flowering responses in photoperiod-sensitive cannabis cultivars (Schilling et al., 2023) [7]. Moreover, this protocol allows different cultivars to be grown concurrently in the same environment, further contributing to its almost ubiquitous use in the industry. However, the photoperiod responses of modern, indoor-grown cannabis cultivars are not well characterized (Zhang et al., 2021) [1]. The hybridization of genotypes with various photoperiod responses has likely given rise to some modern cannabis cultivars with intermediate (i.e., >12 h) photoperiod responses (Small, 2022) [5], as has been demonstrated in some contemporary studies (e.g., Zhang et al., 2021; Moher et al., 2021; Peterswald et al., 2023; Potter, 2009) [1,8,9,10]. Therefore, it is likely that photoperiods longer than 12 h could be used to transition some indoor-grown cannabis cultivars from vegetative to flowering stages. In so doing, daily crop exposure to photosynthetic light (i.e., daily light integral, DLI) could be increased accordingly, potentially resulting in higher yields (Llewellyn et al., 2022; Rodriguez-Morrison et al., 2021) [11,12]. 

The objective of this study was to investigate the photoperiod responses of multiple indoor-grown cannabis cultivars and model the dynamics of flowering initiation and early inflorescence development. The hypothesis is that some drug-type cannabis cultivars can produce robust flowering responses under photoperiods longer than 12 h, potentially leading to higher yields.

## 2. Materials and Methods

The trial was conducted in a 3 m × 8 m walk-in growth chamber that contained six compartments (each 1.2 m × 1.8 m) separated by white opaque curtains to prevent inter-compartment light contamination higher than 0.05 µmol·m^−2^·s^−1^ (photosynthetic photon flux density, PPFD). The photoperiod responses of indoor-grown cannabis can be extremely sensitive to stray light and night interruption. Zhang et al. (2021) [1] reported delayed or inhibition of cannabis flowering with stray light levels ≥ 2 µmol·m^−2^·s^−1^. We demonstrated that the cannabis cultivar ’Royal Goddess’ experienced delayed flowering responses at localized (i.e., leaf level) light intensities ≤0.1 µmol·m^−2^·s^−1^ (Llewellyn et al., 2022) [13]. Therefore, extreme care is required both to isolate and characterize stray light coming from all directions within experimental plots. For this study, the maximum allowable stray light anywhere within the plant canopy was set at 0.05 µmol·m^−2^·s^−1^. This was accomplished using opaque and light absorbing materials to reduce stray light penetration into each plot and confirmed following Llewellyn et al. (2022) [13] using a light pollution meter (SQ640, Apogee Instruments, Logan, UT, USA). 

In each compartment, two full-spectrum LED fixtures (Jungle–LED G4i 1200, Allstate Garden Supply, Ontario, CA, USA) were hung 135 cm above the top of the growing substrate (i.e., 142 cm above bench level). The LED fixtures were set to 75% of their maximum intensity. Spectrum and photosynthetic photon flux density (PPFD) were measured on a 20 cm × 20 cm grid in each plot (i.e., 63 measurements per compartment) at the top of the substrate using a spectrometer (LI-180, LI-COR Biosciences, Lincoln, NE, USA). The PPFD (average ± SD) was 354 ± 34 µmol·m^−2^·s^−1,^ and uniformity (i.e., minimum PPFD/maximum PPFD) was 0.67. The relative spectral photon flux distribution of the LED fixtures is provided in Figure S1. 

There were six photoperiod treatments randomly assigned to the six compartments: 12 h, 12.5 h, 13 h, 13.5 h, 14 h, and 15 h. Once the photoperiod trial began, the light fixtures in all treatments were turned on daily at 08:00 and turned off at the end of their prescribed photoperiods (e.g., 20:00 for the 12-h treatment, 20:30 for the 12.5-h treatment, etc.) using digital timers (UG-TR/D2/120 Ultra Grow, Allstate Garden Supply). The curtains at the front of each compartment were manually opened each day at 08:00 and closed at 20:00. This practice aimed to maximize environmental uniformity whenever all light fixtures were on and eliminate stray light between plots once individual treatments began turning off. An internet-connected webcam was used to continuously monitor the curtain positions and duty cycles of the lighting in every compartment.

Ten drug-type *Cannabis sativa* cultivars (Table 1), all with typical THC concentrations in dried inflorescence ≥19%, were sourced from a single commercial grower in Ontario, Canada. For each cultivar, 156 uniform stem tip cuttings were taken from vegetative mother plants and inserted into Rockwool plugs (Macroplug, Grodan, Milton, ON, Canada) and rooted in humidity domes under ≈150 µmol·m^−2^·s^−1^ of cool-white fluorescent lighting on an 18-h photoperiod at the cultivator’s facility. All cuttings were taken on 21 January 2022, and the photoperiod treatments were initiated on 16 February 2022. Sixteen days (d) after cloning, uniformly rooted cuttings were delivered to the research facility at the University of Guelph (Guelph, ON, Canada) and inserted into rockwool blocks (7.6 cm × 7.6 cm × 6.6 cm, Grodan). Transplants were acclimated to chamber conditions of 25 °C, 80% relative humidity (RH), ambient CO_2_, and LED lighting (described above) on an 18-h photoperiod (18 h light/6 h dark), with lights off daily between 02:00 and 08:00.

After 10 d of acclimation, 84 uniform-sized plants of each cultivar with an intact primary shoot were selected, subdivided into 6 groups of 14 plants, and randomly assigned to the plots. After distributing all plants, each plot comprised 10 columns (front to back) of 14 plants, with each column containing a single cultivar. The plants were evenly spaced (8.0 cm within columns and 12.0 cm between columns, measured “on center”) on subirrigation trays (4 × 4 ft grow trays, Botanicare, Vancouver, WA, USA). The locations of the columns of each individual cultivar were randomized in each plot. The photoperiod treatments were invoked on this day.

The plants were subirrigated as needed with a vegetative-stage nutrient solution using the recipe prescribed by Zheng (2022) [14] until 9 days after the initiation of photoperiod treatments. After that, the plants were subirrigated, as needed, with a flowering-stage nutrient solution (Zheng, 2022) [14]. Deionized water was used to make up the nutrient solutions, and their pH was adjusted to 5.7.

The chamber temperature was set at a constant 25 °C. A fogging system added humidity whenever the RH dropped below 60% using an environment controller (Titan, Argus Systems, Surrey, BC, Canada), which recorded temperature, relative humidity, and CO_2_ levels every 5 min. The average (±SD) temperature and RH recorded by the controller were 25.0 ± 0.84 °C and 78.4 ± 8.8%, respectively. There was no CO_2_ supplementation, but the ≈15 air changes per hour chamber ventilation rate ensured that CO_2_ concentration remained ≥400 PPM. Radiation-shielded dataloggers (MX2301A) located at canopy level in each plot recorded temperature and humidity levels at 5-min intervals. The aerial environments in the plots during daytime and nighttime periods were reasonably uniform among the treatments (Table S1), indicating that the photoperiod treatments were relatively independent of other environmental parameters.

### 2.1. Data Collection

#### 2.1.1. Flowering Initiation Phase

**Figure 1:**
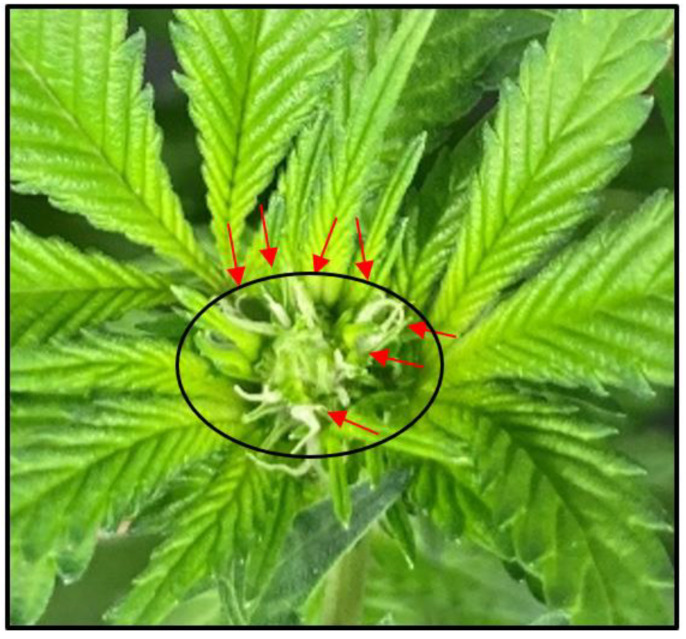
Morphological attributes of flowering initiation at the apex of the primary shoot, approximately 7 days after the plant was identified as having initiated flowering. Each red arrow points to an individual pair of stigmas that, in combination (i.e., ≥3 pairs) and without physical gaps, identify these floral tissues and the surrounding sugar leaves as an inflorescence (circled in black).

Some photoperiodic cannabis cultivars can produce solitary flowers with single pairs of visible stigmas independently from photoperiod responses (Duchin et al., 2020; Spitzer-Rimon et al., 2019; Spitzer-Rimon et al., 2022) [15,16,17]. To eliminate the preexistence of solitary flowers from the assessment of the initiation of the flowering phase, flowering initiation was defined as the appearance of at least three macroscopically visible pairs of stigmas (i.e., an inflorescence) at the apex of the primary shoot (Figure 1). Further, the apical inflorescence is defined as a cluster of 3 or more flowers at the top of the primary shoot with no observable physical gaps between the adjacent flowers (Figure 1). Binary assessments (i.e., yes or no) of elapsed days to flowering initiation (EDTF) assessments were conducted on a per-plant level, commencing 7 d after the start of the photoperiod treatments and lasting for 14 d. Once flowering initiation was identified on a given plant, it was excluded from EDTF assessments on subsequent days. 

#### 2.1.2. Harvest

Immediately after the end of the EDTF assessments, one cultivar was harvested per day according to the harvest schedule in Table 1. Before cutting each plant at the top of the substrate, height (i.e., substrate surface to the tallest part of the plant) and two widths (i.e., widest part of the plant and perpendicular to this) were recorded. The growth index (GI) was calculated using the formula: (height × width_1_ × width_2_)/300 (Ruter, 1992) [18]. The most representative plant from each cultivar by photoperiod treatment combination was defoliated prior to cutting, and all six plants from each treatment were photographed together. The apical inflorescence from every plant was removed, and length, diameter, and fresh weight (FW) were recorded. The volume of the apical inflorescence (i.e., apical volume) was estimated using the volume of a cylinder formula: V = L × π × (D/2)^2^. In this formula, V is volume (mm^3^), L is length (mm), and D is diameter (mm). All remaining floral tissues were then stripped from each plant, and their combined FW was recorded. All remaining aboveground tissues (i.e., stems, branches, and leaves) were combined, and aboveground vegetative FW was recorded. The floral tissues of three representative plants from each cultivar by photoperiod treatment combination were oven-dried to a constant weight at 70 °C and reweighed. The water content of the floral tissues was calculated using the formula: (fresh weight − dry weight)/fresh weight × 100%. Harvest index (HI) was calculated using the formula: total floral FW/(total floral FW + aboveground vegetative FW). All plant tissue weights were measured using a digital balance (BCE2202-1S; Sartorius Lab Instruments, Göttingen, Germany) and reported in grams (g), according to normal conventions.

#### 2.1.3. Statistical Analysis

There was good inter- and intra-cultivar uniformity in plant size at the start of the trial (data not shown). However, due to the combination of different growth habits (e.g., indica- vs. sativa-dominant), relatively low light intensity, high planting density, and randomized cultivar positioning within each compartment, there was some inhibition of growth for certain cultivars in specific compartments during the later stages of the trial. In cases where relatively weak plants were adjacent to more robust plants, these plants may have either failed to initiate flowering or failed to thrive beyond the end of the EDTF assessment phase, and sometimes they died. These plants were removed from the analyses either at the end of the EDTF assessment or at harvest using the outlier detection protocol described below. Hence, the number of replicate values for each measured parameter varied between the cultivar by photoperiod treatment combinations (Tables S2 and S3). Outliers for each measured parameter were detected and removed on a cultivar–treatment basis using the interquartile range exclusive method in Excel (Office v2302, Microsoft, Redmond, DC, USA). For every individual plant, an outlier in any one harvest parameter would remove the plant from all of the harvest parameters. However, outliers in the EDTF assessment were not excluded from the harvest parameters. This is because some plants did not initiate flowering but did continue to produce vegetative biomass within normal ranges until they were harvested. Quadratic and linear regressions were conducted for each measured parameter in each cultivar using statistical analysis software (Rstudio; v2021.9.0.351, Boston, MA, USA). Photoperiod was used as the continuous, independent variable.

The best-fit models were selected based on the *p*-values of the respective models’ coefficients. The Akaike Information Criterion (AIC) values were compared between linear and quadratic models for each cultivar by parameter combination. If AIC_linear_ − AIC_quadratic_ ≥ 2.0 and the second-order coefficient of the quadratic model were significant (*p* ≤ 0.05), then the quadratic model was chosen. If the quadratic model was not chosen and the first-order coefficient of the linear model was significant (*p* ≤ 0.05), then the residuals were examined for heteroskedasticity. If heteroskedasticity was present, the dependent variable was log-transformed using the natural logarithm as the base. No model was chosen if neither the second-order coefficient of the quadratic model nor the first-order coefficient of the linear model were significant. The *p*-values and R^2^ for the best-fit models of each cultivar by parameter combination are presented in Tables S4–S8.

## 3. Results

Images of defoliated whole plants and the apical region of the primary shoot of representative plants from each photoperiod treatment are provided in Figures S2–S4 for BD, GG, and PD, respectively. These images illustrate the flowering initiation, plant growth, and early inflorescence development responses of the ten cultivars to the photoperiod treatments, which are described in the following sections.

Depending on the slope direction (i.e., positive in EDTF and negative in harvest parameters), both linear and log-linear responses are generally indicative of the optimum photoperiod being ≈12 h for each individual parameter and cultivar combination. In contrast, for cultivar by parameter combinations that responded quadratically to photoperiod, the vertices of the respective models are indicative of ideal photoperiods that may be greater than 12 h. The determination of a true “optimum” photoperiod will depend on the origins and growth habits of individual cultivars and cultivators’ specific production strategies and growing environments.

### 3.1. Initiation of Flowering Stage

**Figure 2:**
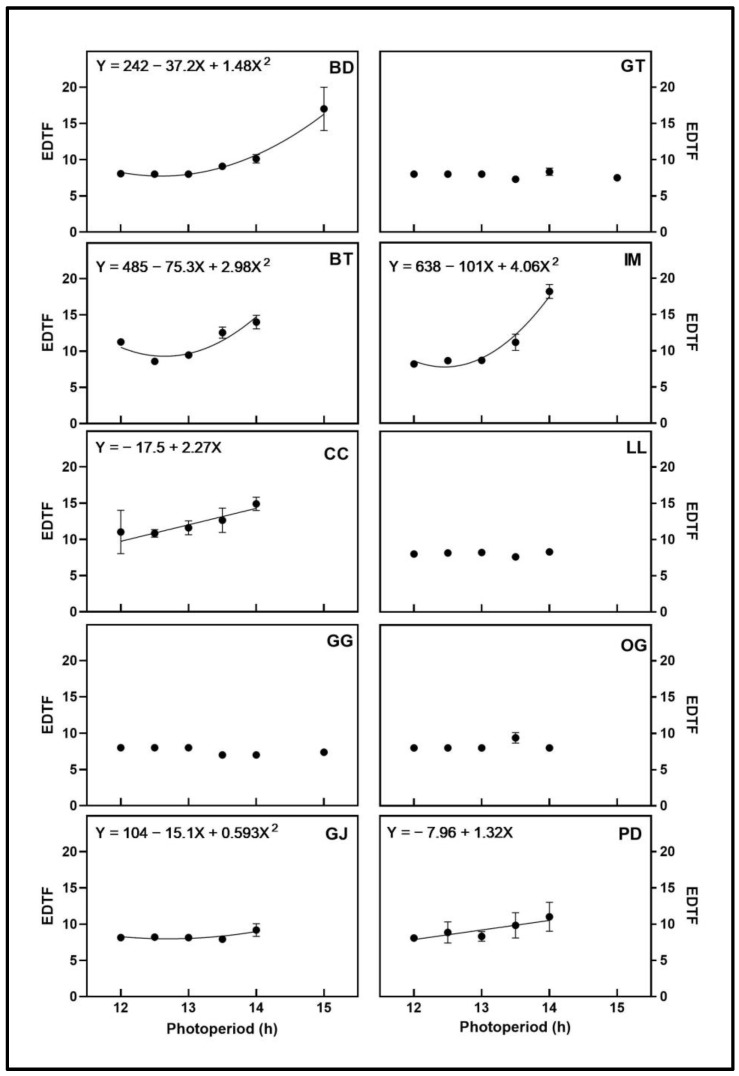
Elapsed days to flowering (EDTF) responses of ten cannabis cultivars to different photoperiod treatments (12 h, 12.5 h, 13 h, 13.5 h, 14 h, and 15 h). Each point represents the mean of that treatment ± SE. When error bars are not visible, they are smaller than the respective symbols. The curves represent the best-fit models for each cultivar; equations for these models are also provided. When no curve is present, there was no significant relationship between photoperiod and EDTF. Where there was no initiation of flowering in the 15-h treatment in some cultivars, no data are shown for this treatment. Cultivars are: ‘Blue Dream’ (BD), ‘Ghost Train Haze’ (GT), ‘Black Triangle’ (BT), ‘Incredible Milk’ (IM), ‘Chem de la Chem’ (CC), ‘Legendary Larry’ (LL), ‘Gorilla Glue’ (GG), ‘OG Kush’ (OG), ‘Garlic Jelly’ (GJ), ‘Powdered Donuts’ (PD).

Elapsed days to flowering (EDTF) represents the time between the switch from the 18-h photoperiod to the individual photoperiod treatments and the visible appearance of at least three pairs of stigmas at the top of the primary shoot on a given plant (i.e., inflorescence initiation). There were no photoperiod treatment effects on EDTF in GG and GT; all treatments initiated flowering at approximately 8 d (Figure 2). There were no photoperiod treatment effects on EDTF between 12 h and 14 h in LL and OG, at approximately 8 d, but there were no signs of flowering initiation in the 15-h treatment. The EDTF in CC and PD increased linearly with increasing photoperiod between 12 h and 14 h, but no plants in either cultivar initiated flowering in the 15-h treatment. The EDTF of four cultivars (BD, BT, GJ, and IM) responded quadratically to increasing photoperiod, with minima at 12.6 h, 12.6 h, 12.7 h, and 12.4 h, respectively. Blue Dream (BD) was the only cultivar with photoperiod treatment effects on EDTF but initiated flowering in the 15-h treatment. None of the floral tissues that initiated in the 15-h treatment continued to develop beyond the initiation phase. Therefore, the floral biomass results presented in the following sections do not include data from the 15-h treatment. For cultivars whose EDTF did not respond to photoperiod, photoperiods from 12 to 14 h (LL and OG) or from 12 to 15 h (GG and GT) may be considered the optimum period for this parameter. For cultivars that responded linearly (CC and PD) to increasing photoperiod, 12 h may be considered the optimum photoperiod. For the cultivars (BD, BT, GJ, and IM) that responded quadratically to increasing photoperiod, the vertices of their responses may be indicative of optimum photoperiods greater than 12 h for initiating flowering.

### 3.2. Apical Inflorescence Size

**Figure 3:**
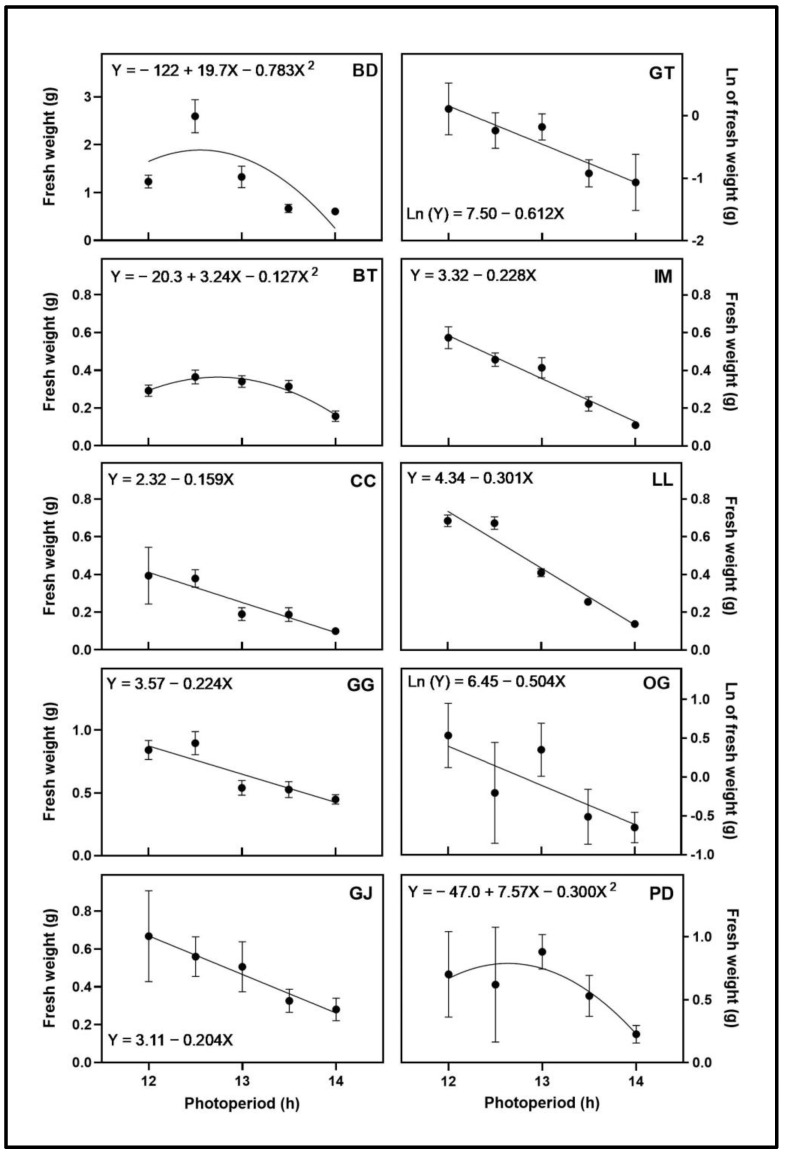
Apical inflorescence fresh weight responses of ten cannabis cultivars to different photoperiod treatments (12 h, 12.5 h, 13 h, 13.5 h, and 14 h). Each point represents the mean of that treatment ± SE. When error bars are not visible, they are smaller than the respective symbols. The curves represent the best-fit models for each cultivar; equations for these models are also provided. Cultivars are: ‘Blue Dream’ (BD), ‘Ghost Train Haze’ (GT), ‘Black Triangle’ (BT), ‘Incredible Milk’ (IM), ‘Chem de la Chem’ (CC), ‘Legendary Larry’ (LL), ‘Gorilla Glue’ (GG), ‘OG Kush’ (OG), ‘Garlic Jelly’ (GJ), ‘Powdered Donuts’ (PD).

**Figure 4:**
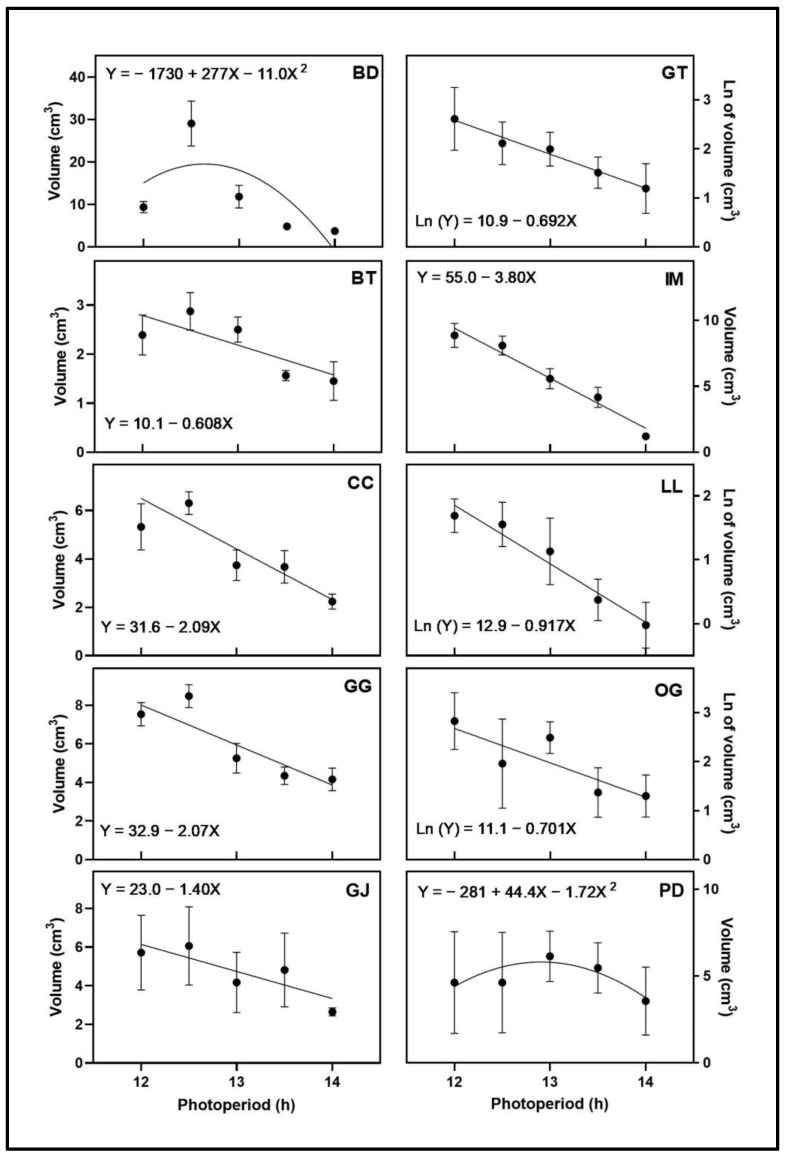
Apical inflorescence volume responses of ten cannabis cultivars to increasing photoperiod treatments (12 h, 12.5 h, 13 h, 13.5 h, and 14 h). Each point represents the mean of that treatment ± SE. When error bars are not visible, they are smaller than the respective symbols. The curves represent the best-fit models for each cultivar; equations for these models are also provided. Cultivars are: ‘Blue Dream’ (BD), ‘Ghost Train Haze’ (GT), ‘Black Triangle’ (BT), ‘Incredible Milk’ (IM), ‘Chem de la Chem’ (CC), ‘Legendary Larry’ (LL), ‘Gorilla Glue’ (GG), ‘OG Kush’ (OG), ‘Garlic Jelly’ (GJ), ‘Powdered Donuts’ (PD).

The apical inflorescence FW (Figure 3) and volume (Figure 4) responses to photoperiod varied by cultivar. While plants from the 15-h photoperiod treatment in BD, GG, and GT all showed signs of initiation of flowering during the first 3 weeks of the trial, these did not develop further. The apical inflorescence FW and volume of BD and PD responded quadratically to photoperiod, with maxima at 12.6 h for both cultivars. The maxima of apical inflorescence volume for BD and PD were at 12.6 h and 12.9 h, respectively. The apical inflorescence FW and volume of CC, GG, GJ, IM, and LL all decreased linearly with increasing photoperiod. The natural log of the apical inflorescence FW and volume of GT and OG decreased with increasing photoperiod. The natural log of the apical inflorescence volume for LL decreased with increasing photoperiod. The only cultivar with different types of regression models for apical inflorescence FW and volume was BT. The apical inflorescence FW responded quadratically to photoperiod, with a maximum at 12.8 h, and the apical inflorescence volume decreased linearly with increasing photoperiod.

### 3.3. Total Floral Biomass

There were no photoperiod treatment effects on inflorescence water content for any cultivars. This indicates that floral FW measurements were appropriate for making floral biomass comparisons between treatments (Table 2). The water content of the harvested floral biomass ranged between 79% and 83%, depending on the cultivar.

**Figure 5:**
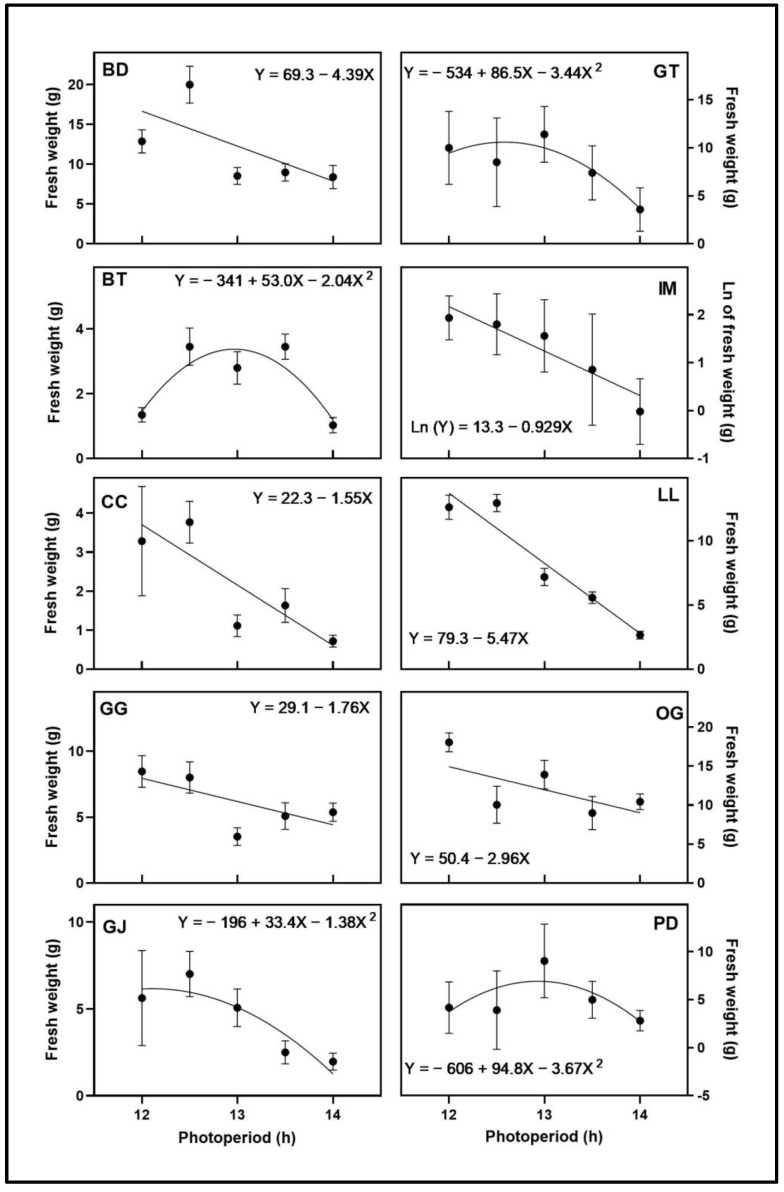
Total floral fresh weight responses of ten cannabis cultivars to different photoperiod treatments (12 h, 12.5 h, 13 h, 13.5 h, and 14 h). Each point represents the mean of that treatment ± SE. When error bars are not visible, they are smaller than the respective symbols. The curves represent the best-fit models for each cultivar; equations for these models are also provided. Cultivars are: ‘Blue Dream’ (BD), ‘Ghost Train Haze’ (GT), ‘Black Triangle’ (BT), ‘Incredible Milk’ (IM), ‘Chem de la Chem’ (CC), ‘Legendary Larry’ (LL), ‘Gorilla Glue’ (GG), ‘OG Kush’ (OG), ‘Garlic Jelly’ (GJ), ‘Powdered Donuts’ (PD).

Total floral FW of BD, CC, GG, LL, and OG decreased linearly with increasing photoperiod (Figure 5). The log of total floral FW of IM decreased with increasing photoperiod. The total floral FW of BT, GJ, GT, and PD responded quadratically to photoperiod, with maxima at 13.0 h, 12.1 h, 12.6 h, and 12.9 h, respectively.

### 3.4. Harvest Index

**Figure 6:**
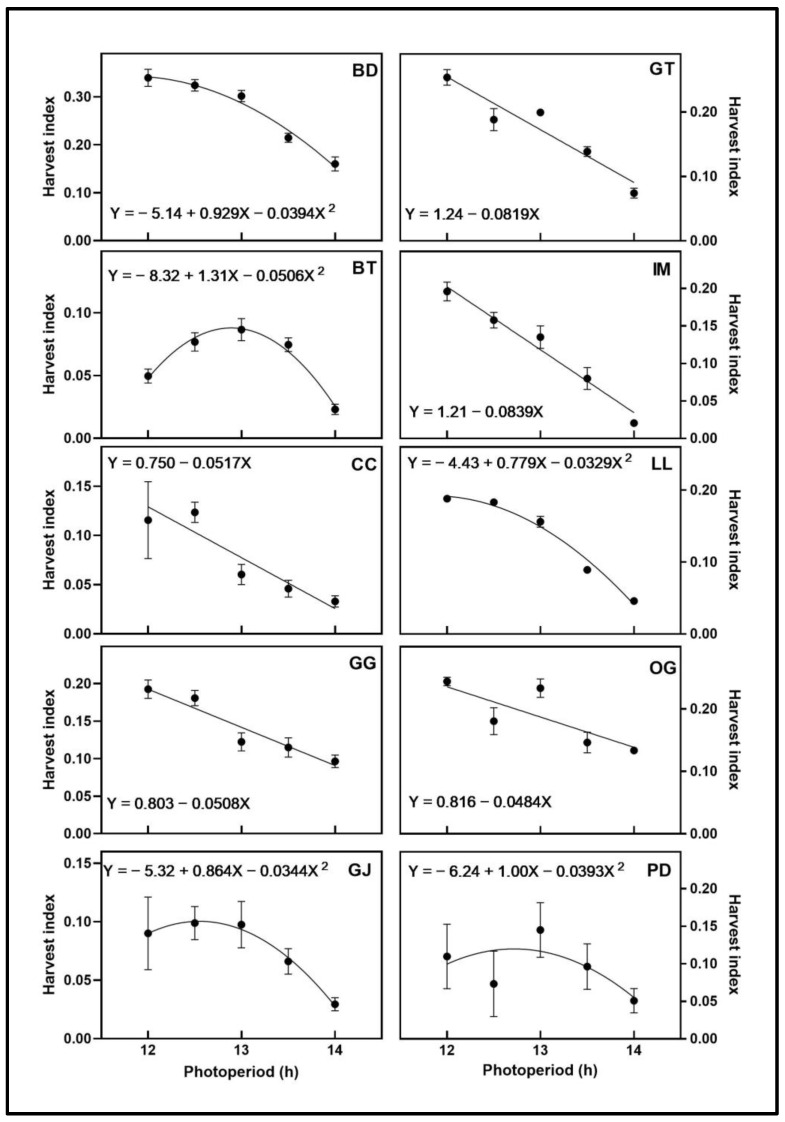
Harvest index (total floral fresh weight/total plant fresh weight × 100) responses of ten cannabis cultivars to different photoperiod treatments (12 h, 12.5 h, 13 h, 13.5 h, and 14 h). Each point represents the mean of that treatment ± SE. When error bars are not visible, they are smaller than the respective symbols. The curves represent the best-fit models for each cultivar; equations for these models are also provided. Cultivars are: ‘Blue Dream’ (BD), ‘Ghost Train Haze’ (GT), ‘Black Triangle’ (BT), ‘Incredible Milk’ (IM), ‘Chem de la Chem’ (CC), ‘Legendary Larry’ (LL), ‘Gorilla Glue’ (GG), ‘OG Kush’ (OG), ‘Garlic Jelly’ (GJ), ‘Powdered Donuts’ (PD).

The harvest index of BD, BT, GJ, LL, and PD responded quadratically to photoperiod with maxima at 11.8 h, 12.9 h, 12.3 h, 11.8 h, and 12.7 h, respectively (Figure 6). The harvest index of CC, GG, GT, IM, and OG decreased linearly with increasing photoperiod.

## 4. Discussion

Invoking a 12-h photoperiod after a period of vegetative growth under long days (i.e., ≥16 h light/≤8 h dark) is the predominant protocol for transitioning from vegetative to reproductive growth stages in indoor cannabis production (Zheng and Llewellyn, 2022; Potter, 2014) [6,19]. While this approach starkly contrasts the photoperiod dynamics in natural environments, it is highly efficacious in provoking robust flowering initiation responses in indoor-grown cannabis. Schilling et al. (2023) [7] showed that this photoperiod protocol could both accelerate and standardize the flowering initiation responses of cultivars with widely ranging natural photoperiod responses. Further, hastening the transition from vegetative to reproductive growth could increase the potential for maximizing inflorescence yield at commercial maturity and/or minimizing the length of the production cycle. However, this may be an oversimplification of the actual biomass allocation dynamics between foliar and floral tissues during the entirety of the flowering stage, including the production of secondary metabolites. Such a short photoperiod constrains the daily PAR exposure to only half of the entire flowering stage of production, imposing hard limits on crop photosynthetic productivity (for a given PPFD). For example, Schilling et al. (2023) [7] noted that seed yields were relatively low under these conditions, and Peterswald et al. (2023) [9] reported substantially lower floral yields under 12-h vs. 14-h photoperiods. With the objective of examining how ideal the 12-h photoperiod really is for optimizing the transition from vegetative to reproductive growth in indoor-grown cannabis, we investigated the flowering initiation and early stage biomass allocation responses of 10 cultivars to photoperiods ranging from 12 h to 15 h.

Evaluating the flowering initiation time in cannabis is not straightforward. The morphological parameters used to identify when a cannabis plant has initiated flowering have been inconsistent among published studies or have been left ambiguous by some authors. Additionally, floral primordia and even solitary flowers (i.e., with macroscopically visible pairs of stigmas) have been observed on plants growing in long-day (i.e., ≥16 h) conditions (e.g., from personal observations and in Spitzer-Rimon et al. (2019) [16]). Solitary flowers may be especially common in clonally propagated plants, which is a prevalent propagation method used by commercial indoor cannabis cultivators. Depending on the age and health of the mother plants, the physiological age of the propagules used in clonal propagation may be substantially older than seed-propagated cannabis at the time of flowering. Juvenile seed-propagated plants may have a physiological age requirement before they can flower, and clonally propagated plants can have macroscopically visible floral primordia prior to being excised from the mothers (Spitzer-Rimon et al., 2022) [17]. Therefore, we focused on the rapidly growing region at the apex of the primary shoot to characterize flowering initiation in the present study, ensuring that flowering was being evaluated only on new tissues (i.e., those that developed under their respective photoperiod treatments). Utilizing a cluster containing ≥3 pairs of macroscopically visible stigmas (i.e., an inflorescence) as the minimum criterion further reduced the risk of previously-developed or solitary flowers obscuring the EDTF assessments.

Despite removing outliers, the photoperiod responses of some parameter by cultivar combinations still had relatively high variability. This is evident from the spread of individual data points around the predicted means (i.e., low *R*^2^-values). While a low *R*^2^ may be interpreted as a higher level of uncertainty about the true magnitude of a response or a predicted optimum value, it does not obviate the statistical significance (i.e., *p*-values) of the responses predicted by the types and shapes of the best-fit models. Since modeling flowering initiation and early inflorescence development was the main objective of this study, we chose to maximize the number of observational units for each cultivar by photoperiod combination. However, this approach may have resulted in increases in intra-treatment variability.

We found four cultivars (BD, BT, IM, and GJ) that initiated flowering most rapidly when grown under photoperiods between 12.4 h and 12.7 h. Additionally, we found four cultivars (GT, LL, GG, and OG) with no delay in flowering initiation up to 14 h. Furthermore, two cultivars (CC and PD) demonstrated linear increases in EDTF with increasing photoperiod, but the delays were only 1.3 d and 2.3 d, respectively, at 13 h vs. 12 h. In addition, three cultivars (BD, GT, and GG) initiated flowering under all photoperiod treatments, with only one of these cultivars (BD) having delayed flowering initiation under photoperiods longer than 13 h. Similarly, Moher et al. (2021) [8] reported minimal delays in flowering initiation in explants (i.e., small plants grown in tissue culture) of a drug-type cannabis cultivar grown under 13.2 h vs. 12 h, but substantial delays (or no flowering) under photoperiods ≥13.6 h. However, how cannabis flowering responses in tissue culture relate to larger plants in commercial production has not yet been characterized. Peterswald et al. (2023) [9] also reported little to no delay in flowering initiation in all three tested cannabis cultivars under 14 h vs. 12 h. Furthermore, in 15 essential oil cultivars of hemp, Zhang et al. (2021) [1] reported no delays, relative to 12 h, in flowering initiation at 12.5 h (with at least three cultivars exhibiting no delay up to 13.5 h). However, 1-d to 2-d delays between 13 h and 13.75 h and approximately 10-d delays were noted at 14 h when EDTF was averaged across all cultivars. Zhang et al. (2021) [1] also reported substantial cultivar-dependent variability in EDTF, ranging between approximately 14 d and 26 d, with as little as 0.25-h differences in photoperiod significantly impacting EDTF in some cultivars. Many other studies on hemp have also shown insignificant or only minor delays in flowering initiation under photoperiods of up to 14 h (Hall et al., 2014; Amaducci et al., 2008; Borthwick and Scully, 1954; Lisson et al., 2000; Stack et al., 2021) [4,20,21,22,23]. While these findings suggest that photoperiods longer than 12 h may provoke flowering initiation responses in indoor-grown cannabis, they also collectively illustrate the cultivar-specificity of cannabis’ photoperiod responses.

Keeping in mind that the plants in the present study were grown at a very high planting density (relative to commercial indoor cultivation) and harvested well before commercial inflorescence maturity, the photoperiod responses of the harvest parameters also varied considerably between the cultivars. While flowering had initiated in three cultivars under 15 h (BD, GT, and GG), they did not continue to develop beyond the flowering initiation stage. The phenomenon of flowering initiation under long photoperiods (even up to 24 h) but with no further development also exists in hemp cultivars (Lisson et al., 2000) [22]. This suggests that flowering initiation may be a facultative photoperiod response while inflorescence development is more of an obligate photoperiodic response (Potter, 2009; Thomas and Vince-Prue, 1997) [10,24]. Contrasting with the 15-h treatment, floral tissues of all cultivars in the 14-h treatment continued to develop beyond the initiation stage into inflorescences. However, the floral tissues were generally smaller and less well developed than in plants under the shorter photoperiod treatments (e.g., Figures S2–S4). Whether the floral yield of plants in the 14-h treatment could have caught up to or even surpassed shorter photoperiod treatments [as in Peterswald et al. (2023) [9]] during the remainder of a normal flowering stage (e.g., ≥4 more weeks) requires further investigation.

The harvest index (HI), which portrays the relative biomass allocation between vegetative and reproductive (i.e., floral) tissues, ranged from approximately 0.05 to 0.3, depending on the cultivar and photoperiod. Three cultivars (BT, GJ, and PD) all had maximum HI below 0.1 (i.e., <10% floral biomass). In comparison, the HI in three other cultivars (BD, GT, and OG) was higher than 0.2 in some treatments, illustrating considerable phenotypic variability in floral tissue development during the early part of the flowering stage. Five cultivars had negative linear responses for both floral biomass and HI to increasing photoperiod. When all of these responses are combined, they indicate that both vegetative and total aboveground biomass increased with increasing photoperiod in these cultivars. This is potentially due to the inherently higher DLIs under longer photoperiods. Since vegetative growth of indoor-grown cannabis normally virtually ceases after four to five weeks under a 12-h photoperiod (Peterswald et al., 2023; Potter, 2014; Yep et al., 2020) [9,19,25], the HI of all tested cultivars should increase substantially during the remainder of the flowering stage (Llewellyn et al., 2022; Rodriguez-Morrison et al., 2021; Potter, 2014) [11,12,19]. However, this cessation of vegetative growth (e.g., of photosynthetic tissues) may also confer a “capacity limit” for plants to convert available PAR into marketable biomass during the rest of their life cycle. Therefore, if longer photoperiods promoted additional vegetative growth during the early phases of the flowering stage, either by changing the relative amount of vegetative vs. reproductive growth or simply from higher daily light exposure, this could result in higher yields at commercial floral maturity. Within their unique logistical and infrastructure limitations, indoor cannabis growers are urged to investigate the effects of photoperiods longer than 12 h (up to 14 h) on provoking robust flowering responses and increasing yields in their individual cultivars.

The maxima for apical inflorescence FW in three cultivars (BD, BT, and PD), for apical inflorescence volume in two cultivars (BD and PD), and for total floral yield in three cultivars (BT, GT, and PD) were between 12.6 h and 13 h. The remaining cultivars exhibited some reductions in early stage inflorescence growth and yield with increasing photoperiod (i.e., negatively sloped linear or log-linear models), even when there were no delays in flowering initiation. The allocation of resources between vegetative and floral tissues and between apical vs. higher-order inflorescences is likely cultivar-dependent, according to their phenotypic (e.g., indica vs. sativa growth habits) and photoperiod responses. Therefore, early flowering-stage biomass allocation may not be a good predictor for yield and quality responses of mature floral tissues. For example, Peterswald et al. (2023) [9] reported dramatically (≈30%) higher floral yields in all three cultivars under 14-h vs. 12-h photoperiod treatments. They speculated that the inherently higher daily light integrals (DLI) in longer photoperiod treatments (i.e., 16.7% higher DLI in 14 h vs. 12 h for a given PPFD) contributed to yield increases. This agrees with studies that have shown very strong biomass responses in cannabis to increased PAR exposure (Llewellyn et al., 2022; Rodriguez-Morrison et al., 2021; Moher et al., 2022) [11,12,26]. In contrast, all of the cultivars in the present study had maximum (early flowering-stage) floral yields at photoperiods less than 13 h. Under photoperiod treatments longer than 12 h, the combination of inherently higher DLIs and promotion of vegetative growth (i.e., foliage) during the phase of the flowering stage when vegetative growth is most vigorous may result in greater floral and secondary metabolite yields by the time cannabis plants reach commercial maturity (Peterswald et al., 2023) [9].

The present study measured flowering initiation and harvest parameters for only 3–4 weeks of growth under the short-day photoperiod treatments, which is only approximately one-third of the time normally required to reach commercial floral maturity. We have demonstrated that photoperiods moderately longer than 12 h can initiate robust flowering responses in indoor-grown cannabis cultivation. Previous work suggested tissue culture could be used to determine optimal—in terms of the greatest percentage of plants that initiated flowering—photoperiods for many cultivars at once (Moher et al., 2021) [8]. Since tissue-culture facilities are highly specialized and not readily available to many growers, we hypothesized that flowering initiation and early flower growth of small clonal cannabis transplants grown under different photoperiod treatments could be used to predict the optimum flowering-stage photoperiod for a given cultivar. However, our results demonstrated that these methods of determining optimal photoperiod may not be appropriate because they do not fully characterize the potential photoperiod influences on the temporal dynamics of cannabis’ transition from vegetative to reproductive growth. For example, some cultivars may initiate flowering under longer photoperiods (e.g., 15 h) but may not develop large inflorescences; therefore, high yields will not be achieved.

In future investigations, determining the optimum photoperiod for maximizing inflorescence yield and quality of different indoor-grown cannabis cultivars must incorporate the entire flowering stage, from flowering initiation to commercial maturity. This would enable commercially relevant growth and biomass comparisons between photoperiod treatments, including vegetative biomass, floral yield, and secondary metabolite composition of marketable tissues. Because small changes in photoperiod can substantially affect flowering (Zhang et al., 2021) [1], the time gaps between adjacent treatments in future studies should be kept relatively small. According to Zhang et al. (2021) [1] and the results of the current study, future studies should focus on photoperiod treatments within the 12 h to 14 h range. Carefully designed studies incorporating a range of cannabis cultivars and growth habits may also elucidate patterns among phenotypic groups. This would enable cultivators to better predict the photoperiod responses of their individual cultivars. Along with maximizing yield potential, this may also facilitate the development of commercial production protocols whereby cultivars with similar photoperiod responses can be grown concurrently in the same cultivation environment (i.e., grow room).

## 5. Conclusions

This study showed that many cultivars of indoor-grown cannabis are capable of initiating flowering under photoperiods up to 14 h, and some even up to 15 h, with only minor delays in flowering initiation times. However, even in cultivars that experienced no delay in flowering initiation, there were general decreases in apical inflorescence size and early stage floral yield under photoperiods longer than 13 h. Future research should examine the yield and quality (e.g., cannabinoid composition) effects of photoperiods longer than 12 h on indoor-grown cannabis cultivars grown to commercial floral maturity.

## Figures and Tables

**Table 1:** Cultivar abbreviations, indica to sativa ratio, the normal time to maturity, and harvest schedule of each cultivar.

Cultivar Name (Abbreviation)	Indica:Sativa ^z^	Days to Maturity ^y^	Days to Harvest ^x^
Black Triangle (BT)	100:0	56–63	21
Garlic Jelly (GJ)	50:50	60–70	22
Ghost Train Haze (GT)	20:80	65–80	23
Powdered Donuts (PD)	80:20	60–70	24
Chem de la Chem (CC)	20:80	60–66	25
Legendary Larry (LL)	70:30	63–70	26
Gorilla Glue (GG)	60:40	56–63	27
OG Kush (OG)	75:25	60	28
Incredible Milk (IM)	20:80	56–63	29
Blue Dream (BD)	30:70	63	30

^z^ the ratio of indica and sativa phenotypes used when breeding each cultivar, according to our commercial partner. ^y^ number of days between invoking a 12-h photoperiod and commercial inflorescence maturity, according to our commercial partner. ^x^ number of days after initiating photoperiod treatments that plants were harvested in this trial.

**Table 2:** Inflorescence water content (mean ± SD, *n* = 15) for each cannabis cultivar.

Cultivar Name (Abbreviation)	Inflorescence Water Content (% of FW)
Black Triangle (BT)	82 ± 1.3
Garlic Jelly (GJ)	83 ± 0.6
Ghost Train Haze (GT)	81 ± 0.8
Powdered Donuts (PD)	82 ± 0.9
Chem de la Chem (CC)	80 ± 2.0
Legendary Larry (LL)	79 ± 0.9
Gorilla Glue (GG)	82 ± 0.4
OG Kush (OG)	80 ± 0.9
Incredible Milk (IM)	80 ± 1.4
Blue Dream (BD)	79 ± 0.9

## Data Availability

All data from the study are included in the manuscript, further inquiries can be directed to the corresponding author.

## Supplementary Materials

The following supporting information can be downloaded at: https://www.mdpi.com/article/10.3390/plants12142605/s1, Figure S1: Relative spectral photon flux distribution used in all experimental plots, Table S1: Canopy-level mean (±SD) temperatures and relative humidity in each treatment plot during the periods when lights were off (night) and on (day), from the start of the photoperiod treatments to the beginning of harvest, Table S2: The number of plants in each cultivar by photoperiod combination that initiated flowering during the assessment of elapsed days to flowering (EDTF), Table S3: The number of evaluated plants in each cultivar by photoperiod combination in harvest and post harvest plant growth and inflorescence yield parameters, Table S4: Best-fit models and summary statistics for elapsed days to flowering (EDTF) for each *C. sativa* cultivar. Qu = quadratic, Li = linear. No model was selected if *p* > 0.05, Table S5: Best-fit models and summary statistics for the fresh weight of the apical inflorescence for each *C. sativa* cultivar. Qu = quadratic, Li = linear, Lo = log-transformed, Table S6: Best-fit models and summary statistics for the volume of the apical inflorescence for each *C. sativa cultivar*. Qu = quadratic, Li = linear, Lo = log-transformed, Table S7: Best-fit models and summary statistics for total inflorescence fresh weight for each *C. sativa* cultivar. Qu = quadratic, Li = linear, Lo = log-transformed, Table S8: Best-fit models and summary statistics for harvest index for each *C. sativa cultivar*. Qu = quadratic, Li = linear, Figure S2: (a) De-leafed *C. sativa* ’Blue Dream’ (BD) plants of photoperiod treatments (from left to right): 12 h, 12.5 h, 13 h, 13.5 h, 14 h, and 15 h on day 30 after the start of the photoperiod treatments. (b) Apical region of the primary shoot of BD from each photoperiod treatment (from left to right): 12 h, 12.5 h, 13 h, 13.5 h, 14 h, and 15 h on day 30 after the start of the photoperiod treatments. The white scale bars represent 10 cm. Figure S3: (a) De-leafed *C. sativa* ‘Gorilla Glue’ (GG) plants of photoperiod treatments (from left to right): 12 h, 12.5 h, 13 h, 13.5 h, 14 h, and 15 h on day 27 after the start of the photoperiod treatments. (b) Apical region of the primary shoot of GG from each photoperiod treatment (from left to right): 12 h, 12.5 h, 13 h, 13.5 h, 14 h, and 15 h on day 27 after the start of the photoperiod treatments. The white scale bars represent 10 cm, Figure S4: (a) De-leafed *C. sativa* ’Powdered Donuts’ (PD) plants of photoperiod treatments (from left to right): 12 h, 12.5 h, 13 h, 13.5 h, 14 h, and 15 h on day 24 after the start of the photoperiod treatments. (b) Apical region of the primary shoot of PD from each photoperiod treatment (from left to right): 12 h, 12.5 h, 13 h, 13.5 h, 14 h, and 15 h on day 24 after the start of the photoperiod treatments. The white scale bars represent 10 cm.
