# Supplementary material for: Is Twelve Hours Really the Optimum Photoperiod for Promoting Flowering in Indoor-Grown Cultivars of Cannabis sativa?
Source: Plants (Basel). 2023 Jul 10;12(14):2605. doi: 10.3390/plants12142605 (PMC10386198; doi:10.3390/plants12142605)
Supplement: Supplementary file 1 [file plants-12-02605-s001.zip › plants-2358750-supplementary.pdf]

Supplementary Figures and Tables

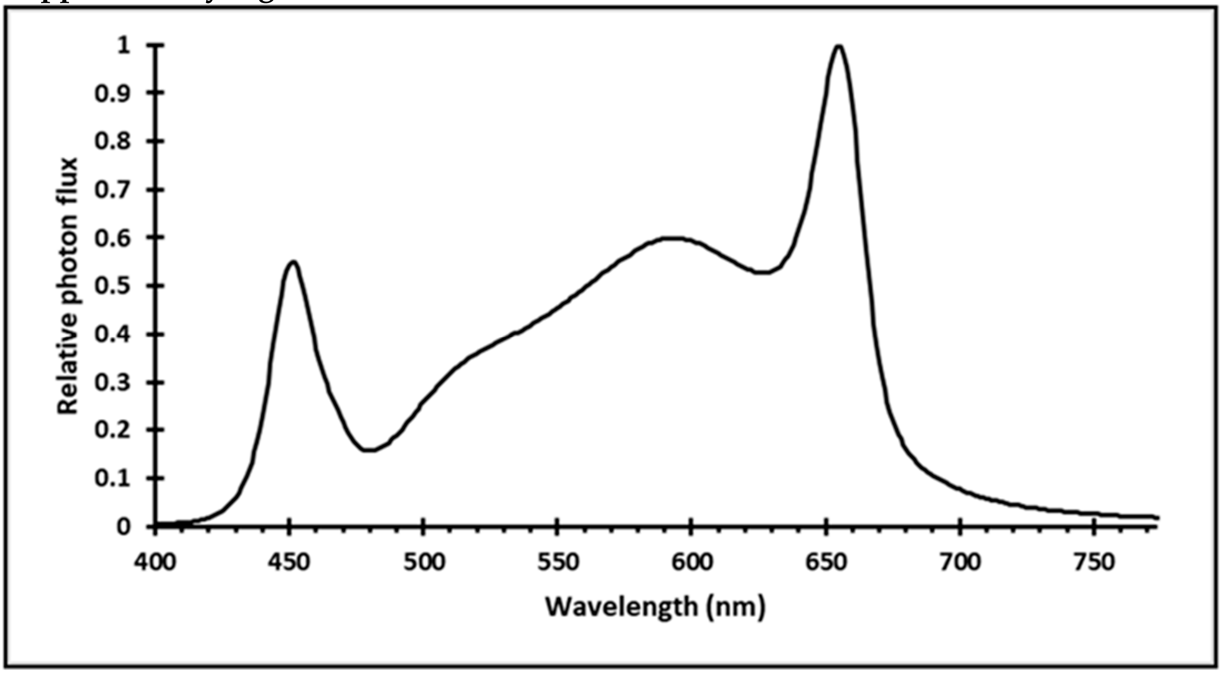

Figure S1. Relative spectral photon flux distribution used in all experimental plots.

Table S1. Canopy-level mean (± SD) temperatures and relative humidity in each treatment plot during the periods when lights were off (night) and on (day), from the start of the photoperiod treatments to the beginning of harvest.

Photoperiod	Lights	Temperature (°C)	Relative Humidity (%)
12.0 h	Night	24.0 ± 0.41	77.5 ± 8.07
	Day	25.7 ± 0.75	81.3 ± 6.20
12.5 h	Night	24.0 ± 0.40	78.9 ± 7.78
	Day	25.7 ± .073	83.2 ± 5.77
13.0 h	Night	24.4 ± 0.34	74.2 ± 7.12
	Day	25.1 ± 0.52	81.1 ± 5.85
13.5 h	Night	24.4 ± 0.35	76.1 ± 7.46
	Day	25.5 ± 0.52	82.2 ± 5.92
14.0 h	Night	24.4 ± 0.33	75.4 ± 6.84
	Day	25.2 ± 0.46	82.3 ± 5.56
15.0 h	Night	24.5 ± 0.75	77.4 ± 6.82
	Day	25.4 ± 0.66	83.9 ± 5.97

Table S2. The number of plants in each cultivar by photoperiod combination that initiated flowering during the assessment of elapsed days to flowering (EDTF).

Cultivar	Photoperiod					
	12 h	12.5 h	13 h	13.5 h	14 h	15 h
Black Triangle (BT)	8	14	13	13	9	0
Garlic Jelly (GJ)	14	14	13	12	11	0
Ghost Train Haze (GT)	14	11	14	14	12	14
Powdered Donuts (PD)	13	7	10	12	8	0
Chem de la Chem (CC)	3	11	12	8	9	0
Legendary Larry (LL)	14	14	14	10	14	0
Gorilla Glue (GG)	14	14	13	14	13	13
OG Kush (OG)	13	11	12	13	11	0
Incredible Milk (IM)	12	13	9	7	6	0
Blue Dream (BD)	14	14	14	14	9	2

Table S3. The number of evaluated plants in each cultivar by photoperiod combination in harvest and post harvest plant growth and inflorescence yield parameters.

Cultivar	Photoperiod				
	12 h	12.5 h	13 h	13.5 h	14 h
Black Triangle (BT)	10	9	13	8	8
Garlic Jelly (GJ)	11	11	9	11	8
Ghost Train Haze (GT)	13	10	13	13	11
Powdered Donuts (PD)	10	6	7	8	8
Chem de la Chem (CC)	3	10	11	12	13
Legendary Larry (LL)	11	11	8	9	11
Gorilla Glue (GG)	12	9	9	9	12
OG Kush (OG)	7	11	10	9	7
Incredible Milk (IM)	11	12	10	8	7
Blue Dream (BD)	9	12	7	9	8

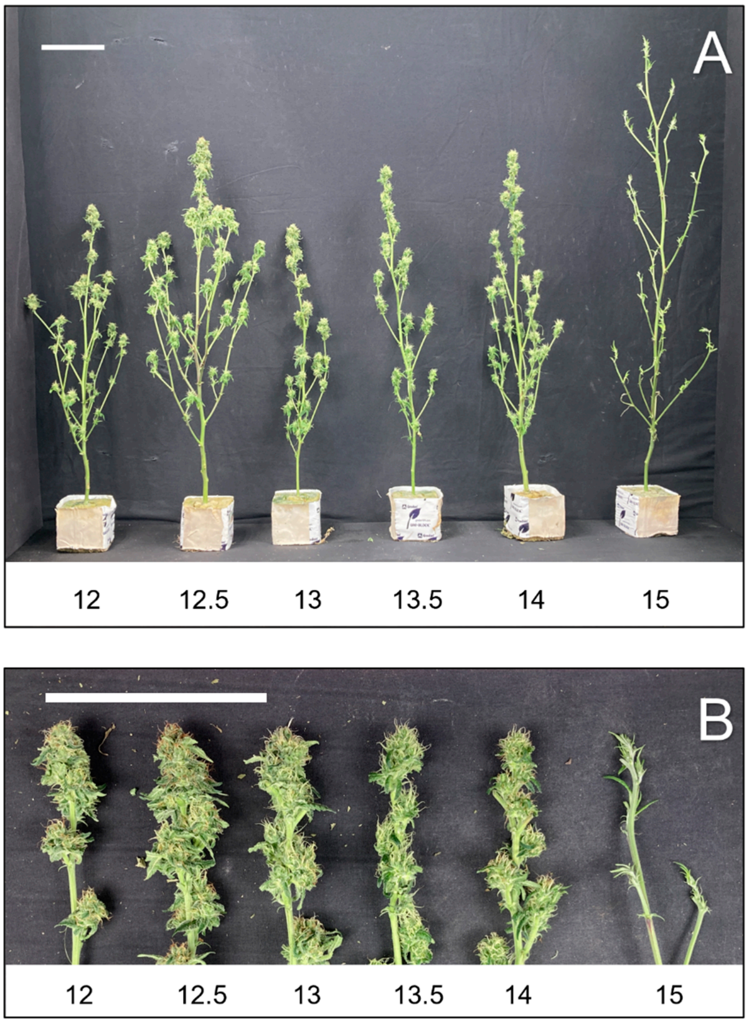

Figure S2. (a) De-leafed *C. sativa* ‘Blue Dream’ (BD) plants of photoperiod treatments (from left to right) 12 h, 12.5 h, 13 h, 13.5 h, 14 h, and 15 h on day 30 after the start of the photoperiod treatments. (b) Apical region of the primary shoot of BD from each photoperiod treatment (from left to right) 12 h, 12.5 h, 13 h, 13.5 h, 14 h, and 15 h on day 30 after the start of the photoperiod treatments. The white scale bars represent 10 cm.

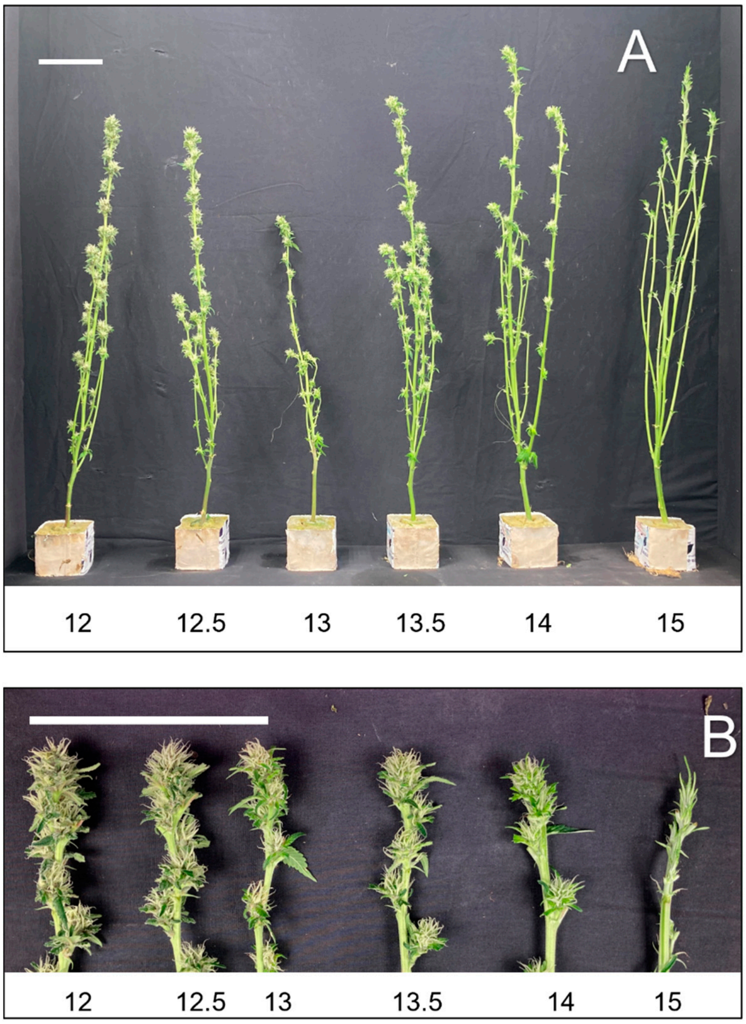

Figure S3. (a) De-leafed *C. sativa* 'Gorilla Glue' (GG) plants of photoperiod treatments (from left to right) 12 h, 12.5 h, 13 h, 13.5 h, 14 h, and 15 h on day 27 after the start of the photoperiod treatments. (b) Apical region of the primary shoot of GG from each photoperiod treatment (from left to right) 12 h, 12.5 h, 13 h, 13.5 h, 14 h, and 15 h on day 27 after the start of the photoperiod treatments. The white scale bars represent 10 cm.

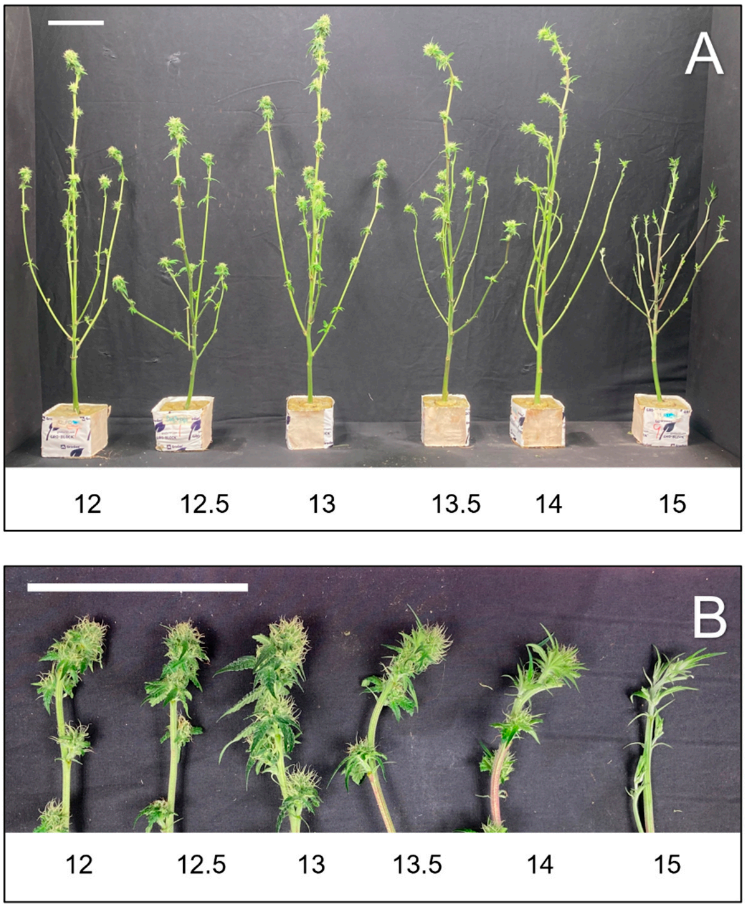

Figure S4. (a) De-leafed *C. sativa* 'Powdered Donuts' (PD) plants of photoperiod treatments (from left to right) 12 h, 12.5 h, 13 h, 13.5 h, 14 h, and 15 h on day 24 after the start of the photoperiod treatments. (b) Apical region of the primary shoot of PD from each photoperiod treatment (from left to right) 12 h, 12.5 h, 13 h, 13.5 h, 14 h, and 15 h on day 24 after the start of the photoperiod treatments. The white scale bars represent 10 cm.

Table S4. Best fit models and summary statistics for elapsed days to flowering (EDTF) for each *C. sativa* cultivar. Qu = quadratic, Li = linear. No model was selected if $P > 0.05$.

Cultivar	Best fit model (Qu, Li)	*p*-value	R^2
Blue Dream (BD)	Qu	< 0.0001	0.70
Black Triangle (BT)	Qu	< 0.0001	0.48
Chem de la Chem (CC)	Li	0.0076	0.16
Gorilla Glue (GG)	none	-	-
Garlic Jelly (GJ)	Qu	0.00060	0.29
Ghost Train Haze (GT)	none	-	-
Incredible Milk (IM)	Qu	< 0.0001	0.79
Legendary Larry (LL)	none	-	-
OG Kush (OG)	none	-	-
Powdered Donuts (PD)	Li	< 0.0001	0.33

Table S5. Best fit models and summary statistics for the fresh weight of the apical inflorescence for each *C. sativa* cultivar. Qu = quadratic, Li = linear, Lo = log transformed.

Cultivar	Best fit model (Qu, Li, Lo)	*p*-value	R^2
Blue Dream (BD)	Qu	0.020	0.32
Black Triangle (BT)	Qu	0.00042	0.33
Chem de la Chem (CC)	Li	< 0.0001	0.40
Gorilla Glue (GG)	Li	< 0.0001	0.38
Garlic Jelly (GJ)	Li	< 0.0001	0.52
Ghost Train Haze (GT)	Lo	< 0.0001	0.60
Incredible Milk (IM)	Li	< 0.0001	0.56
Legendary Larry (LL)	Li	< 0.0001	0.86
OG Kush (OG)	Lo	0.00012	0.30
Powdered Donuts (PD)	Qu	0.0067	0.36

Table S6. Best fit models and summary statistics for the volume of the apical inflorescence for each *C. sativa* cultivar. Qu = quadratic, Li = linear, Lo = log transformed.

Cultivar	Best fit model (Qu, Li, Lo)	*p*-value	R^2
Blue Dream (BD)	Qu	0.021	0.26
Black Triangle (BT)	Li	0.0088	0.14
Chem de la Chem (CC)	Li	< 0.0001	0.34
Gorilla Glue (GG)	Li	< 0.0001	0.37
Garlic Jelly (GJ)	Li	0.00034	0.24
Ghost Train Haze (GT)	Lo	< 0.0001	0.54
Incredible Milk (IM)	Li	< 0.0001	0.56
Legendary Larry (LL)	Lo	< 0.0001	0.77
OG Kush (OG)	Lo	< 0.0001	0.33
Powdered Donuts (PD)	Qu	0.049	0.36

Table S7. Best fit models and summary statistics for total inflorescence fresh weight for each *C. sativa* cultivar. Qu = quadratic, Li = linear, Lo = log transformed.

Cultivar	Best fit model (Qu, Li, Lo)	*p*-value	R^2
Blue Dream (BD)	Li	0.0022	0.20
Black Triangle (BT)	Qu	0.00010	0.29
Chem de la Chem (CC)	Li	< 0.0001	0.33
Gorilla Glue (GG)	Li	0.0069	0.14
Garlic Jelly (GJ)	Qu	0.025	0.52
Ghost Train Haze (GT)	Qu	0.028	0.33
Incredible Milk (IM)	Lo	< 0.0001	0.42
Legendary Larry (LL)	Li	< 0.0001	0.75
OG Kush (OG)	Li	0.045	0.09
Powdered Donuts (PD)	Qu	0.0033	0.22

Table S8. Best fit models and summary statistics for harvest index for each *C. sativa* cultivar. Qu = quadratic, Li = linear.

Cultivar	Best fit model (Qu, Li)	*p*-value	R^2
Blue Dream (BD)	Qu	0.013	0.74
Black Triangle (BT)	Qu	< 0.0001	0.52
Chem de la Chem (CC)	Li	< 0.0001	0.48
Gorilla Glue (GG)	Li	< 0.0001	0.53
Garlic Jelly (GJ)	Qu	< 0.0001	0.64
Ghost Train Haze (GT)	Li	< 0.0001	0.70
Incredible Milk (IM)	Li	< 0.0001	0.70
Legendary Larry (LL)	Qu	< 0.0001	0.92
OG Kush (OG)	Li	0.00043	0.26
Powdered Donuts (PD)	Qu	0.016	0.24
